# Interrelated Effects of Zinc Deficiency and the Microbiome on Group B Streptococcal Vaginal Colonization

**DOI:** 10.1128/msphere.00264-22

**Published:** 2022-08-09

**Authors:** Lindsey R. Burcham, Zachary M. Burcham, Madeline S. Akbari, Jessica L. Metcalf, Kelly S. Doran

**Affiliations:** a Department of Immunology and Microbiology, University of Colorado School of Medicine, Aurora, Colorado, USA; b Department of Animal Science, Colorado State University, Fort Collins, Colorado, USA; c Department of Microbiology, University of Tennessee, Knoxville, Tennessee, USA; University of Michigan-Ann Arbor

**Keywords:** vaginal colonization, dietary zinc, vaginal microbiome, *Streptococcus agalactiae*, group B *Streptococcus*, GBS, *Akkermansia muciniphila*

## Abstract

Group B Streptococcus (GBS) in the vaginal tract is a risk factor for preterm birth and adverse pregnancy outcomes. GBS colonization is also transient in nature, which likely reflects the contributions of pathogen determinants, interactions with commensal flora, and host factors, making this environment particularly challenging to understand. Additionally, dietary zinc deficiency is a health concern on the global scale that is known to be associated with recurrent bacterial infection and increased rate of preterm birth or stillbirth. However, the impact of zinc deficiency on vaginal health has not yet been studied. Here we use a murine model to assess the role of dietary zinc on GBS burden and the impact of GBS colonization on the vaginal microbiome. We show that GBS vaginal colonization is increased in a zinc-deficient host and that the presence of GBS significantly alters the microbial community structure of the vagina. Using machine learning approaches, we show that vaginal community turnover during GBS colonization is driven by computationally predictable changes in key taxa, including several organisms not previously described in the context of the vaginal microbiota, such as Akkermansia muciniphila. We observed that *A. muciniphila* increases GBS vaginal persistence and, in a cohort of human vaginal microbiome samples collected throughout pregnancy, we observed an increased prevalence of codetection of GBS and *A. muciniphila* in patients who delivered preterm compared to those who delivered at full term. These findings reveal the importance and complexity of both host zinc availability and native microbiome to GBS vaginal persistence.

**IMPORTANCE** The presence of group B Streptococcus (GBS) in the vaginal tract, perturbations in the vaginal microbiota, and dietary zinc deficiency are three factors that are independently known to be associated with increased risk of adverse pregnancy outcomes. Here, we developed an experimental mouse model to assess the impact of dietary zinc deficiency on GBS vaginal burden and persistence and to determine how changes in GBS colonization impact vaginal microbial structure. We have employed unique animal, *in silica* metabolic, and machine learning models, paired with analyses of human cohort data, to identify taxonomic biomarkers that contribute to host susceptibility to GBS vaginal persistence. Collectively, the data reported here identify that both dietary zinc deficiency and the presence of *A. muciniphila* could perpetuate an increased GBS burden and prolonged exposure in the vaginal tract, which potentiate the risk of invasive infection *in utero* and in the newborn.

## INTRODUCTION

Vaginal colonization by Streptococcus agalactiae, or group B Streptococcus (GBS), serves as an infectious reservoir, increasing the potential for invasive infection in pregnant mothers, developing fetuses, and newborn babies. To reduce the risk of neonatal GBS infection after birth, the CDC recommends screening of late-term pregnant mothers and intravenous delivery of antibiotics for those testing positive at the onset of labor (1); however, there are currently no preventative interventions in place to protect the pregnant mother or developing fetus from GBS infection during pregnancy. Further, higher GBS bacterial burden and prolonged exposure in the reproductive tract lead to increased fetal risk and likelihood of adverse pregnancy outcomes (2). It is therefore imperative that we understand more about the factors contributing to GBS vaginal colonization to reduce maternal, fetal, and neonatal GBS exposure.

One factor intricately associated with susceptibility to prenatal infection is malnutrition (3). Zinc deficiency, specifically due to dietary limitations, affects an estimated 80% of pregnant women worldwide (4) and is strongly associated with adverse pregnancy outcomes including labor and delivery complications and an increased risk for the development of infections (5–9). Few studies have aimed to elucidate the molecular mechanisms of zinc uptake in GBS (10–12); however, a gap remains in the understanding of how nutrient zinc deficiency impacts reproductive tract pathogenesis, including GBS vaginal colonization and persistence, or how this could impact the vaginal microbial community structure. We sought to characterize the effects of dietary zinc deficiency on the vaginal microbiome and GBS colonization in the vaginal tract using unique combined *in vivo* and *in silico* approaches.

Here we report that zinc-deficient mice have increased GBS bacterial burden in the vaginal tract and that the structure of the vaginal microbial community is altered throughout the course of GBS colonization. We show that longitudinal changes in the vaginal microbiota occur in a computationally predictable manner due to changes in several key taxa that were previously undescribed in the context of the vaginal microbiome. Further, we identified the bacterial species Akkermansia muciniphila to have the potential for metabolic cross-feeding with GBS *in silico* and to impact GBS persistence in a murine model. Finally, in a human cohort of vaginal microbiome data, *A. muciniphila* and GBS were detected at increased frequency in patients whose pregnancies resulted in a preterm birth.

## RESULTS

### Impact of murine zinc deficiency on GBS vaginal colonization.

**FIG 1:**
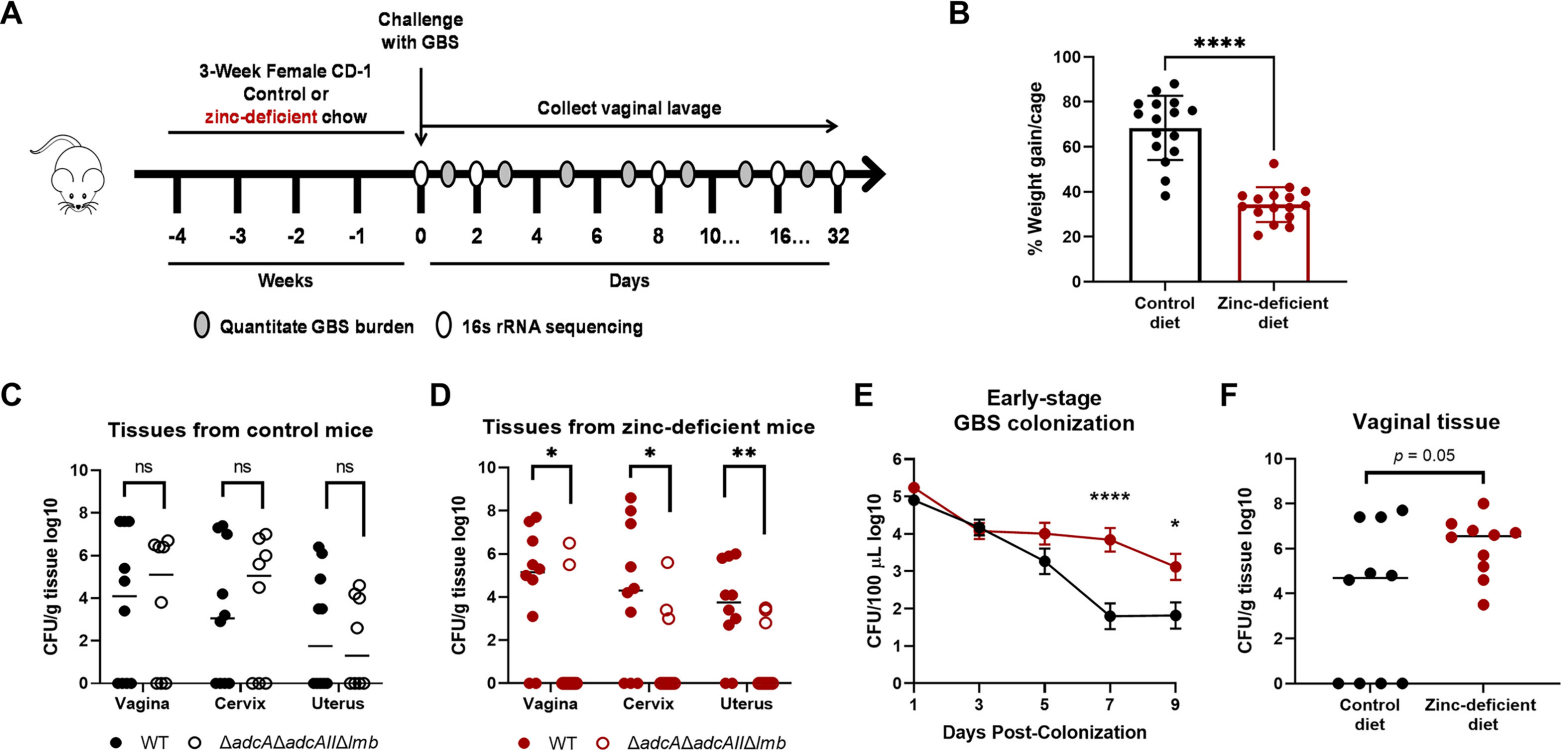
Establishment and assessment of a murine model of dietary zinc deficiency during vaginal colonization. (A) Murine model of dietary zinc deficiency. (B) Weight gain of mice fed a control or zinc-deficient diet. (C and D) Confirmation of control (C) or dietary zinc deficiency (D) using GBS zinc-transport mutant *in vivo*. (E and F) GBS vaginal lumen colonization (E) and vaginal tissue GBS burden (F) during early stage colonization. Significance was determined by unpaired student’s two-tailed *t* test (B to D and F) and two-way ANOVA with Sídák’s multiple-comparison test (E), with *, *P < *0.05; **, *P < *0.01; ****, *P < *0.0001; ns, not significant.

To determine how host zinc status impacts GBS vaginal colonization and vaginal microbial communities, we adapted previous murine models of dietary zinc deficiency (13, 14) and paired this with our established model of GBS vaginal colonization (15). Three-week-old female CD-1 outbred mice were randomly separated and fed either a control (29 ppm Zn) or zinc-deficient (0 ppm Zn) chow for 4 weeks (Fig. 1A). We monitored all mice for dietary intake and weight gain throughout the 4 weeks and observed a significant decrease in weight gain in the zinc-deficient mice compared to the control fed mice (Fig. 1B). To confirm dietary zinc deficiency, control and zinc-deficient mice were challenged intravaginally with GBS wild type (WT) or a mutant lacking the three substrate-binding proteins responsible for maintaining GBS intracellular zinc homeostasis, Δ*adcAΔadcAII*Δ*lmb* (10–12). No differences were observed for GBS burden in the reproductive tissues between the WT and the Δ*adcAΔadcAII*Δ*lmb* strains in control mice (Fig. 1C); however, the Δ*adcAΔadcAII*Δ*lmb* mutant strain was not able to colonize vaginal tissue or ascend to the higher reproductive tissues of zinc-deficient mice (Fig. 1D), demonstrating a difference of zinc bioavailability in reproductive tract tissues. To assess GBS vaginal colonization and persistence, and the effect of GBS and dietary zinc deficiency on the vaginal microbiome, mice were synchronized in estrus on day −1 and on day 0, prior to GBS inoculation, we collected vaginal lavage to profile the naive vaginal microbiota (Fig. 1A). Throughout the course of colonization, we collected vaginal lavage samples daily for either quantification of GBS burden or assessment of vaginal microbiota (Fig. 1A). We consistently recovered increased GBS CFU from the vaginal lumen in zinc-deficient mice during early stage colonization (Fig. 1E). Moderate increases in GBS burden were confirmed in the vaginal tissues of zinc-deficient mice compared to tissues from control mice harvested on day 7 postcolonization (Fig. 1F). Beginning at day 15 postcolonization, mice from both groups began to clear GBS and maintained similarly low levels of GBS vaginal colonization until the experimental endpoint (Fig. S1A in the supplemental material).

10.1128/msphere.00264-22.1 FIG S1 (A) GBS bacterial burden in the vaginal lumen during late-stage colonization. (B) PCoA of unweighted UniFrac distances with 95% confidence intervals of vaginal microbiome communities prior to GBS colonization and colored by diet type. Download FIG S1, TIF file, 0.6 MB. Copyright © 2022 Burcham et al. 2022 Burcham et al. https://creativecommons.org/licenses/by/4.0/ This content is distributed under the terms of the Creative Commons Attribution 4.0 International license.

### GBS colonization impacts murine community state type and vaginal microbial diversity.

**FIG 2:**
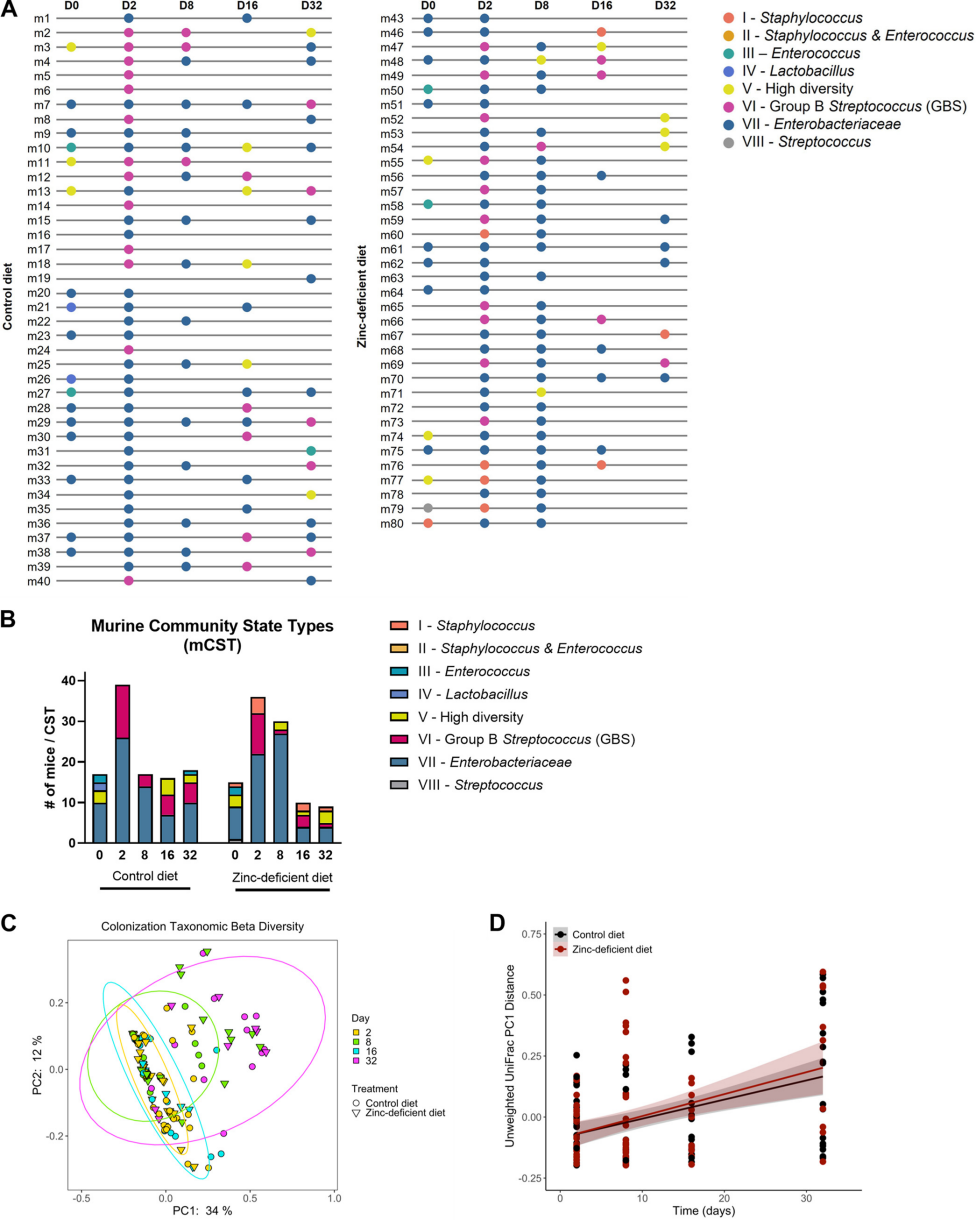
GBS colonization impacts mCST and the vaginal microbiome community. (A and B) Longitudinal assessment of mCSTs of mice prior to and throughout the course of GBS colonization. D, day. (C) Principal coordinate analyses (PCoA) of unweighted UniFrac distances with 95% confidence intervals colored by day and shaped by treatment. (D) Linear mixed-effects model of unweighted UniFrac principal coordinate axis 1 change over time and between treatment.

Vaginal lavage samples collected on days 0, 2, 8, 16, and 32 were processed for 16S rRNA gene amplicon sequencing to profile the initial vaginal microbiota prior to the introduction of GBS and to longitudinally assess changes throughout GBS colonization and after clearance. For initial observations on day 0, the murine vaginal communities did not differ based on diet type (unweighted UniFrac; permutational multivariate analysis of variance: *n* = 33, pseudo-F = 1.95, *P* = 0.081, permutations = 999) (Fig. S1B). However, we identified a significant difference in the detection of unclassified Streptococcus between mice fed a control or zinc-deficient diet (analysis of composition of microbiomes [ANCOM] W = 112), although this difference did not persist after the introduction of GBS. We next assessed murine community state types (mCST) using previously described mCST I-VI groupings (16, 17). In contrast to what was observed previously for mCSTs in C57BL/6 mice, we observed 56% of our naive CD-1 outbred mice (18/32), prior to the introduction of GBS, to have a vaginal community dominated by *Enterobacteriaceae*, which we have named here as mCST VII (Fig. 2A and B). Within this group, we observed a large percentage to be Proteus sp. dominant (Fig. 2A and B). We also noted the rare emergence of mice colonized predominantly with *Streptococci* not classified as GBS and have named this mCST VIII. The original classification of mCST V included mice colonized with a mixture of taxa and a high diversity index (17). In comparing the mCST distribution of mice across diets, we observed a loss of the mCST IV, an emergence of the mCST I, and an increase in representation of the mCST VII in zinc-deficient mice compared to mice fed the control diet (Fig. 2B).

We then assessed the changes in diversity within and between vaginal communities following the introduction of GBS. Community changes were visualized by principal coordinate analyses and statistically tested using linear mixed-effects (LME) models. The unweighted UniFrac distances derived from the first axis of the principal coordinate analysis were responsible for 34% of all the data variation and demonstrated that communities became more phylogenetically dissimilar over time (Pr>|z| < 0.001) with no difference between diet (Pr>|z| = 0.957) or interaction between time and diet (Pr>|z| = 0.634) (Fig. 2C and D; Table S1 and S2). Weighted UniFrac distances did not significantly change over time or treatment (Table S1 and S2). The difference of significance between UniFrac distance metrics is indicative of the importance of lower abundance taxa in driving the changes in diversity since weighted measurements skew toward abundant taxa while unweighted measurements consider only presence or absence data that skew toward the rarer taxa present (18). Analysis of taxonomic richness (i.e., number of unique microbial features) showed an increase of features over time (Pr>|z| < 0.001) independent of diet (Pr>|z| = 0.723), with no interaction between time and diet (Pr>|z| = 0.471) (Tables S1 and S2). Factoring in evenness of microbial features with Shannon’s diversity showed no significant difference over time or between treatments (Table S1 and S2).

10.1128/msphere.00264-22.3 TABLE S1 Linear mixed-effects model summaries for alpha and beta diversities and for centered-log abundance of temporally important species. #, Model did not converge. Download Table S1, XLSX file, 0.01 MB. Copyright © 2022 Burcham et al. 2022 Burcham et al. https://creativecommons.org/licenses/by/4.0/ This content is distributed under the terms of the Creative Commons Attribution 4.0 International license.

10.1128/msphere.00264-22.4 TABLE S2 Linear mixed-effects model results and statistics for alpha and beta diversities and for centered-log abundance of temporally important species. #, Model did not converge. Download Table S2, XLSX file, 0.01 MB. Copyright © 2022 Burcham et al. 2022 Burcham et al. https://creativecommons.org/licenses/by/4.0/ This content is distributed under the terms of the Creative Commons Attribution 4.0 International license.

### GBS colonization alters key vaginal community taxa in a computationally predictable manner.

**FIG 3:**
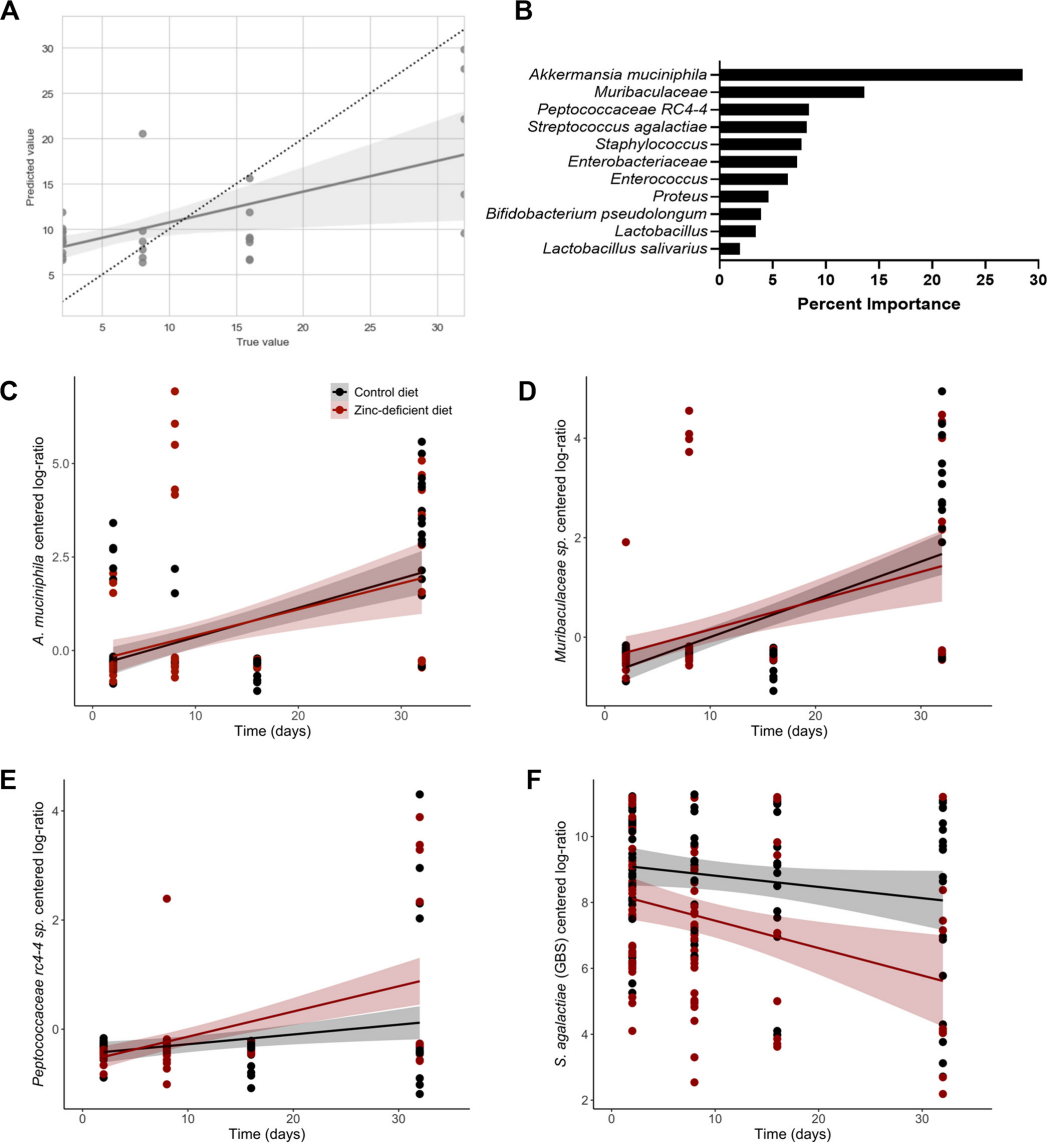
Random Forest machine learning models predict key taxa of the vaginal microbiota that drive diversity throughout GBS colonization. (A) Random forest regressor accuracy for predicting time using normalized species abundance. (B) The most important species for the random forest regressor to predict time. (C to F) Linear mixed-effects models for measuring the impacts of time and treatment on the CLR-transformed abundances of *A. muciniphila* (C), *Muribaculaceae* sp. (D), *Peptococcaceae rc4-4* sp. (E), and S. agalactiae (GBS) (F).

We next sought to determine if the introduction of GBS into the vaginal tract led to predictable changes in the members of the vaginal microbial community over time, using a supervised machine learning approach. In brief, to determine if a significant longitudinal structure exists within the data set, the rarefied species abundances were used to predict the sampling day throughout GBS colonization (days 2 to 32). Changes in the species’ rarefied abundances were found to be significantly predictive of the postinoculation day of GBS colonization (*P* = 3.6e10^−5^, *R*^2^ = 0.4, mean squared error [MSE] = 64.975, SE = 0.0714, m [slope] = 0.339, y-intercept = 7.354) (Fig. 3A). The machine learning algorithm identified 17 species that were informative in predicting day of GBS colonization and ranked them according to their importance in driving the success of the model (Fig. 3B). The changes in rarefied abundance of the top 3 species represented 50.5% of the important information needed for the prediction power of this model. These species were Akkermansia muciniphila (28.5%), an unclassified *Muribaculaceae* (formally *Bacteroidales* S24-7 family) species (13.6%), and an unclassified *Peptococcaceae rc4-4* species (8.4%) (Fig. 3B). LME models were used to measure the significance of all important species’ centered log-ratio (CLR) transformed abundance change over time in relation to diet type (Table S1 and S2, Fig. S2). Interestingly, *A. muciniphila* and *Muribaculaceae* sp. ratios significantly increased over time (Pr>|z| < 0.001, Pr>|z| < 0.001) but independent of diet (Pr>|z| = 0.679, Pr>|z| = 0.157) and with no interaction between diet and time (Pr>|z| = 0.715, Pr>|z| = 0.279) (Fig. 3C and D; Table S1 and S2). *Peptococcaceae rc4-4* sp. ratios significantly increased over time (Pr>|z| = 0.007) with a significant interaction with diet (Pr>|z| = 0.009), demonstrating that *Peptococcaceae rc4-4* sp. ratios in zinc-deficient mice increased at a faster rate than mice fed the control diet (Fig. 3E; Table S1 and S2). GBS ratios trended downwards but were not considered to significantly change over time (Pr>|z| = 0.104), were not different between diets (Pr>|z| = 0.068) and showed no interaction between time and diet (Pr>|z| = 0.13) (Fig. 3F; Table S1 and S2). While we previously identified GBS burden to be significantly higher in zinc-deficient mice and decrease over time by quantifying CFU counts, these results suggest that the ratio of GBS in the community is not significantly impacted throughout the course of colonization. This could be due to (i) GBS and other members decreasing in burden at similar rates or (ii) other non-GBS community members fluctuating in either increasing or decreasing abundance while the GBS to overall community ratio maintains constant. The significant ratio increases observed in *A. muciniphila*, *Muribaculaceae* sp., and *Peptococcaceae rc4-4* sp. suggest the second scenario is more likely to be occurring in this environment (Table S1 and S2). Alternatively, experimental artifacts such as sampling from different stages of estrus or DNA sequenced from dead organisms could impact measurements of GBS burden throughout colonization.

### *A. muciniphila* and GBS may synergize *in vivo* and could prolong host exposure to GBS.

**FIG 4:**
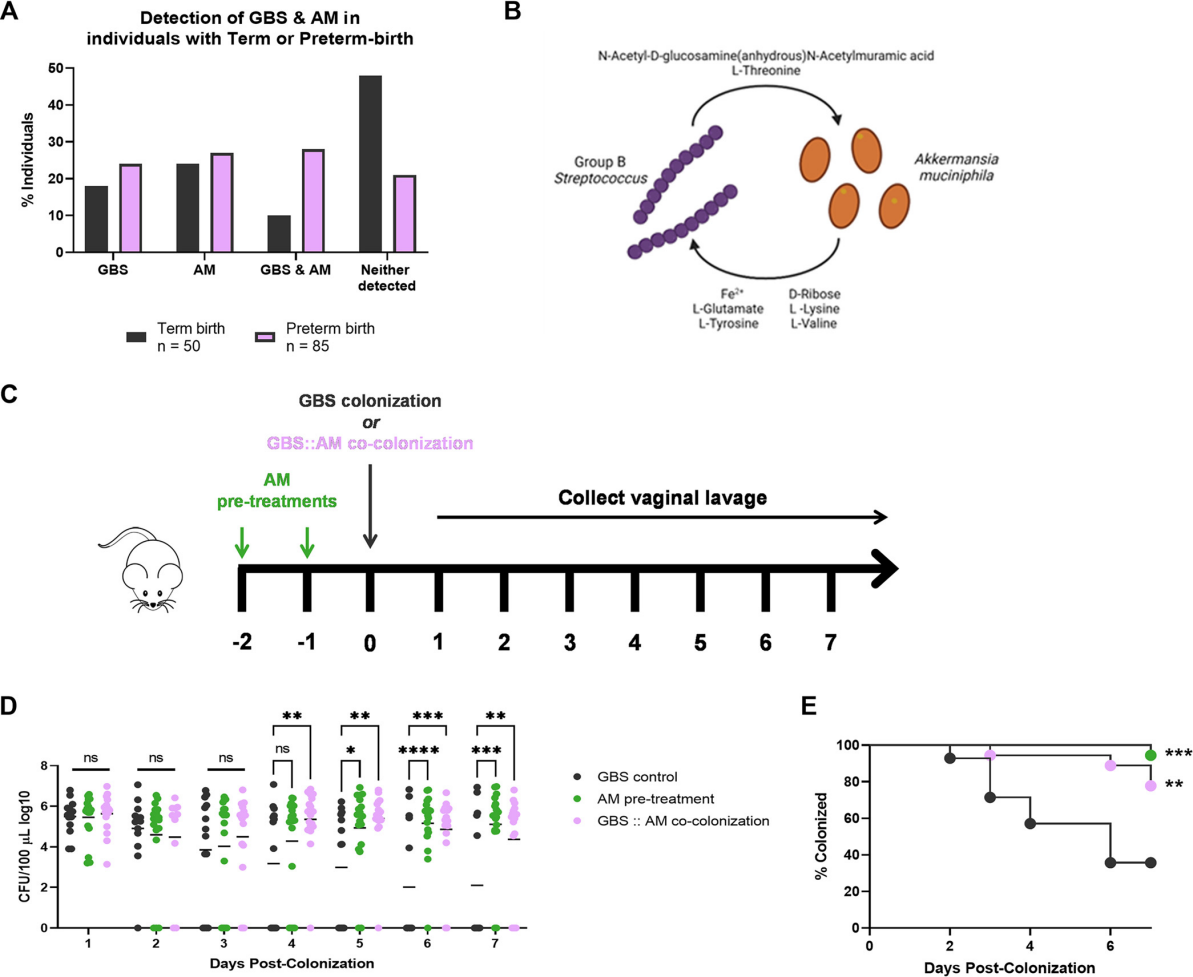
Deciphering the impact of GBS-*A. muciniphila* (AM) interactions in murine and human vaginal health. (A) Detection of GBS, *A. muciniphila*, GBS and *A. muciniphila*, or neither in a human cohort of vaginal microbiome data from term or preterm births. (B) Cross-feeding direction of the eight metabolites most confidently predicted *in silico* to be needed for GBS and *A. muciniphila* cosurvival. (C) Adapted vaginal colonization model to assess GBS and *A. muciniphila* interactions *in vivo.* (D and E) GBS burden (D) and percent (E) of mice colonized with GBS in combination with *A. muciniphila*. Significance was determined by two-way ANOVA with Sídák’s multiple-comparison test (D) and log-rank test (E), with *, *P < *0.05; **, *P < *0.01; ***; *P < *0.001; ****; *P < *0.0001; ns, not significant.

After identifying *A. muciniphila* as the primary organism driving nearly 30% predictive power of our computational models, we aimed to determine if GBS and *A. muciniphila* are detectable in the human vaginal tract. To investigate this we utilized a publicly available data set from Callahan et al. (19), PNAS 2017. In this study, 2,179 vaginal swabs were collected from women throughout the course of pregnancy for 16S rRNA gene sequencing to determine microbial features that contribute to birth outcome. We assessed and stratified this cohort of patient data by those with detectable GBS only, *A. muciniphila* only, codetection of GBS and *A. muciniphila*, or those who had neither organism detected throughout the course of sampling. Of the individuals who delivered at term, we detected GBS in 18% and *A. muciniphila* in 24% (Fig. 4A), and in the individuals that experienced preterm birth, we observed 24% (+6% from term) to have GBS and 27% (+3% from term) to have *A. muciniphila* (Fig. 4A). We also identified a striking increase in the percentages of individuals with both GBS and *A. muciniphila* detected at some point throughout pregnancy from 10% in individuals delivering at term to 28% (+18% from term) in individuals delivering preterm. Conversely, we observed a robust decrease from 48% in term patients to 21% (−27% from term) in preterm patients in the individuals with neither organism detected (Fig. 4A). These results suggest that the presence of GBS and *A. muciniphila* in the vaginal tract may influence birth outcomes, specifically in those patients who had detectable levels of both GBS and *A. muciniphila* throughout pregnancy.

We next sought to understand the potential interactions between GBS and *A. muciniphila* during colonization. We used the genomes of both organisms to assess the potential for metabolic cross-feeding interactions *in silico* using automated genome-scale metabolic model reconstruction (20, 21). A metric of competition, metabolic resource overlap, was calculated to assess the potential nutrient competition between organisms for resources (22). GBS and *A. muciniphila* were found to have limited metabolic competition potential (metabolic resource overlap [MRO] = 0.57). However, metabolic cooperation potential does exist, as 31 metabolites were marked as potential cross-fed compounds between GBS and *A. muciniphila* (Table S3). Ten metabolites were identified as able to be donated from GBS and received by *A. muciniphila* while the other 21 metabolites could be donated from *A. muciniphila* and received by GBS. Eight metabolites were proposed to be frequently taken up by the community for survival and were given a maximum score (SMETANA score = 1.0) of certainty for their potential cross-feeding interaction (Fig. 4B). Of these, GBS could donate *N*-acetyl-d-glucosamine(anhydrous)*N*-acetylmuramic acid and l-threonine, while *A. muciniphila* could donate Fe^2+^, l-glutamate, l-lysine, d-ribose, l-tyrosine, and l-valine (Fig. 4B), further supporting the likelihood of GBS and *A. muciniphila* coexistence *in vivo*. Since these models provided further support that *A. muciniphila* and GBS could coexist in the same environment, we hypothesized that the presence of *A. muciniphila* would impact the ability of GBS to colonize the vaginal tract. To study the impact of *A. muciniphila* presence on GBS colonization *in vivo*, we used our murine model of vaginal colonization where 8-week-old female CD-1 mice were inoculated with GBS alone, inoculated with *A. muciniphila* prior to GBS, or cocolonized with both GBS and *A. muciniphila* at the same time (Fig. 4C). Following the introduction of GBS, vaginal lavage was collected daily to enumerate GBS burden throughout colonization. We observed a significant increase in GBS persistence in the vaginal lumen over time in mice that were inoculated with *A. muciniphila*, either prior or at the same time as GBS compared to those that were inoculated with GBS alone (Fig. 4D and E). These data further suggest that GBS and *A. muciniphila* are capable of coexisting during vaginal colonization and that the presence of *A. muciniphila* may help promote GBS persistence.

10.1128/msphere.00264-22.5 TABLE S3 Metabolites predicted to be cross fed between GBS and *A. muciniphila* with associated species coupling score (SCS), metabolite uptake score (MUS), metabolite production score (MPS), and SMETANA score. Download Table S3, XLSX file, 0.01 MB. Copyright © 2022 Burcham et al. 2022 Burcham et al. https://creativecommons.org/licenses/by/4.0/ This content is distributed under the terms of the Creative Commons Attribution 4.0 International license.

## DISCUSSION

An important aspect to bacterial survival within a harsh, immunologically active site, such as the vaginal mucosa, is the access to nutrient metal ions. Navigating metal homeostasis is an essential process of bacterial physiology and has been characterized in a variety of microorganisms; however, our knowledge on the role of zinc in GBS reproductive tract colonization remains limited. Additionally, dietary zinc deficiency is known to be associated with recurrent infection and increased rate of preterm birth or stillbirth (9). Our model organism, GBS, colonizes the vaginal lumen but can ascend to cause dangerous perinatal infections of the amniotic cavity. This association between zinc deficiency and adverse pregnancy outcomes led us to hypothesize that zinc deficiency could impact GBS vaginal colonization and persistence, leading to a disruption of the vaginal microbiome. Utilizing a modified murine model of vaginal colonization, we show that zinc-deficient mice have increased GBS bacterial burden in the vaginal lumen and vaginal tissues during early colonization. Further, we show that GBS colonization is associated with significant perturbations in the vaginal microbiome. We found that dietary zinc-deficiency had only minor effects on the microbial community structure, which is supported in part by work from a previous study investigating gut health that showed limiting dietary zinc had no effect on the gut microbiome (13). Previous work in the C57BL/6 mouse background found the murine vaginal microbiome to be largely dominated by single or mixed populations of Staphylococcus and/or *Enterococcus*, *Lactobacillus*, or a high diversity mixed population (17). While interestingly, in this work performed in outbred CD-1 mice, we found more than 50% of naive mice have a vaginal microbiota that was dominated by *Enterobacteriaceae*, and more specifically for many, an unidentified species of Proteus. We also noted the emergence of a Streptococcus-dominant community state type, and we have named these two new state types as mCST VII and VIII, respectively.

Computational models utilizing our longitudinal data set were capable of accurately predicting microbial abundance patterns associated with GBS colonization throughout the course of colonization and identified roughly 42% of model accuracy was driven by organisms involved in mucin degradation, including *A. muciniphila* and *Muribaculaceae* sp. *A. muciniphila* is a Gram-negative organism that has been shown to colonize the gut early in life and increase as we age (23). It has also been associated with improved metabolic health (24), and its abundance is inversely associated with obesity (25). Because of their characteristic role in mucin degradation and utilization, *A. muciniphila* and a species of *Muribaculaceae* were included in an intestinal bacteriotherapy consortium that was shown to limit pathogen access to mucosal sugars and moderately limit gut colonization (26), thus suggesting a protective role in these organisms from pathogen colonization. We report here that the presence of *A. muciniphila* could actually promote GBS colonization of the vaginal tract, which could have a negative impact on vaginal health. This work also describes for the first time, an association of *A. muciniphila* with the vaginal tract as it has primarily been regarded as a commensal of the gut. This could be that the vaginal tract is understudied and that microbes in the gut could potentially seed the vaginal microflora.

Collectively, we report herein that GBS vaginal colonization is increased in a zinc-deficient host and significantly impacts the structure of the vaginal microbiota. Further, potential interactions with members of the microbial community, such as *A. muciniphila*, could contribute to increased GBS persistence in the vagina and impact preterm birth in humans. These data represent two unique factors that could perpetuate an increased bacterial burden or prolonged pathogen exposure, which, particularly in the context of pregnancy, potentiate the risk of GBS invasive disease in the mother, fetus, and neonate. Many important and complex questions remain unanswered, including understanding how interactions between GBS and *A. muciniphila* could influence immune signaling within the reproductive mucosa or how these interactions impact colonization in the context of a zinc-deficient or pregnant host. Importantly, this ongoing work has the potential to continue identifying taxonomic and functional biomarkers that contribute to host susceptibility to GBS.

## MATERIALS AND METHODS

### Bacterial strains and growth conditions.

Streptococcus agalactiae (GBS) isolate CJB111 (serotype V) and CJB111Δ*adcA*Δ*adcAII*Δ*lmb* were cultured in Todd-Hewitt broth at 37°C. Mutant strains were constructed previously (12), and deletion of these genes did not affect growth in nutrient rich Todd-Hewitt broth (12). Akkermansia muciniphila *muc* strain (ATCC BAA-835) was grown in prereduced brain-heart infusion media supplemented with 0.1% porcine gastric mucins at 37°C in a Coy Laboratory Products Type A, vinyl anaerobic chamber, using an atmospheric gas mix of N_2_/CO_2_/H_2_ (85/10/5%) (27).

### *In vivo* model of vaginal colonization.

All animal experiments were conducted under the approval of the Institutional Animal Care and Use Committee (no. 00316) at the University of Colorado Anschutz Medical Campus and performed using accepted veterinary standards. We modified our preestablished murine model of vaginal colonization (15, 28), where 3-week-old female CD-1 mice were fed a control (Dyets 515260) or zinc-deficient (Dyets 515258) diet for 4 weeks. Mice were then synchronized with 17β-estradiol by intraperitoneal injection. Mice were inoculated intravaginally with 1 × 10^7^ CFU/10 μL of GBS. The vaginal lumen was lavaged daily with sterile, nuclease free PBS for 16S rRNA gene sequencing and to quantify GBS CFU burden over time. For experiments assessing interactions of GBS and *A. muciniphila*, mice were synchronized in estrus with 17β-estradiol on day −3. On days −2 and −1, the pretreatment group was inoculated intravaginally with two 1 × 10^7^ CFU/10-μL doses of *A. muciniphila*. On experimental day 0, the GBS control group and the *A. muciniphila* pretreatment groups were inoculated intravaginally with 1 × 10^7^ CFU/10 μL of GBS, while the cocolonization group received a 1:1 dose of GBS and *A. muciniphila* 5 × 10^6^ CFU/5 μL of each strain or 1 × 10^7^ CFU/10 μL total bacterial inoculum.

### DNA extraction, amplification, and 16S rRNA sequencing.

DNA was extracted from murine vaginal lavage samples collected on days 0, 2, 8, 16, and 32 along with extraction negative controls and mock community positive controls using the DNeasy PowerSoil Legacy DNA isolation kit (cat. no. 27000-4-KF). 16S rRNA (rRNA) gene amplicons of the V4 region were generated from extractions using modified 515F-806R primer pairs following the Earth Microbiome Project protocols (29). The V4 region was chosen based on its nearly universal bacterial annotation and availability for alignment in reference databases (30). Amplicons were pooled with equal molarity into a sequencing library and next-generation sequencing was performed on the Illumina MiSeq platform to generate 2 × 250 bp reads at the Colorado State University Genomics Center. Raw sequencing data were uploaded to QIITA (study 13660), an open-source microbial study management platform and public data repository (31) and have been made publicly available at European Nucleotide Archive under the accession no. ERP136638. Microbiome analysis scripts and files are available at https://github.com/Metcalf-Lab/GBScolonization2022.

### Microbiome data preprocessing.

Microbiome data was processed and analyzed with QIIME2 version 2021.2 (32). Paired-end reads were imported into QIIME2, demultiplexed, and merged with VSEARCH (33). Joined reads were quality filtered and denoised with Deblur to generate suboperational taxonomic units (i.e., microbial features) and trimmed to 250 bp (34). The deblur pipeline performs *de novo* chimera filtering using UCHIME as implemented by VSEARCH and rapidly uses error profiles in a sensitive manner to obtain putatively true, high quality biological sequences (33). The Greengenes 13.8 99% operational taxonomic unit (OTU) database was utilized for phylogenetic tree creation with SEPP fragment insertion and for taxonomic assignment with a naïve Bayes classifier trained on the 16S rRNA V4 region (35, 36). The Greengenes database was utilized because it is chimera-checked and tailored for 16S rRNA classification. When 16S rRNA alignment is performed, SILVA and Greengenes map comparable to NCBI (37). SEPP fragment insertion performs a phylogenetic placement technique explicitly designed for 16S rRNA data to obtain improved phylogeny trees (38). Microbial features were filtered out if they were assigned to mitochondria, chloroplast, or not of bacterial origin. Further, features were removed to reduce noise if present less than 10 times in the data set and/or not found in at least 2 samples. After filtering, the data set included 365 samples, 9 positive controls, and 28 negative controls. All negative controls except 1 were below 3,000 read counts demonstrating minimal contamination and successful extraction/sequencing. One negative control had a high read count of 20,389. This negative control taxonomically resembled the mock community positive controls, was next to a positive control on the extraction plate, and did not taxonomically resemble any of the murine samples. Therefore, this control was determined to be a product of cross-contamination from the neighboring positive control and removed from the analysis along with the other controls. The read counts of the murine samples ranged from 9 to 75,321 (mean: 17,401.7; median: 12,628). Samples containing less than 3,000 features were removed from the analysis.

### Longitudinal microbiome analyses.

Rarefaction was performed at a depth of 3,000 sequences to provide even sampling across samples for calculating diversity metrics so as not lose too many samples from a single time point or treatment. The QIIME2 diversity plugin was used to compute the following alpha and beta diversity metrics: Shannon’s diversity index (H), observed features (richness), and unweighted UniFrac distances (32, 39). The QIIME2 longitudinal plugin was used for a feature volatility analysis, which utilizes a supervised learning random forest regressor (estimators = 1,000) with cross-validation (k = 5) and hyperparameter tuning to identify informative species with relative abundances that change over colonization time in a predictive manner (40). Eighty percent of the samples are set aside for training the model, and 20 percent of the samples are used for testing the model. Important species to the model are assigned a score between 0 and where the sum of all important species equals to 1. Linear mixed-effects (LME) models evaluate the contribution of covariates to a single dependent variable and were used to test whether diversity metrics and the centered log-ratio transformed abundances of the longitudinally important species were impacted by colonization time and diet treatment. The response variables (i.e., diversity or relative abundances) were statistically assessed over colonization (days 2 to 32) with treatment as an independent variable (fixed effect) and a random intercept for each individual mouse to account for repeated measures. LME models for diversity metrics were calculated for Shannon’s diversity index (H), observed features (richness), and unweighted UniFrac principal coordinate 1 (PC1) distances. Variables from convergent models with *P* values of <0.05 were considered significant. Significance relationships of the predictor variable with the response variable was determined based on the p-value associated with the z-value of each variable (Pr>|z|). Pr>|z| values less than 0.05 are considered significant.

### Determination of murine community state types.

Microbiome murine community state type (mCST) classifications were based on previous classifications described by Vrbanac et al. (17). In brief, state types are described as Staphylococcus dominant (mCST I), Staphylococcus and *Enterococcus*-dominant (mCST II), *Enterococcus* dominant (mCST III), *Lactobacillus* dominant (mCST IV), not dominated by either Staphylococcus or *Enterococcus* and had high alpha diversities (mCST V), and dominated by GBS (mCST VI). In this study, single organism-dominant state types were determined by a minimum representation of 50% of the community. This study established two new mCSTs that were named mCST VII and mCST VIII and were dominated by *Enterobacteriaceae* and Streptococcus, respectively. The mCST VII included organisms classified as *Enterobacteriaceae*, Morganella morganii, and Proteus sp. Mice characterized as mCST V were designated by at least 10 unique microbial features, a Shannon’s index of ≥1.5, and were not dominated by Staphylococcus and/or *Enterococcus*.

### Community interaction simulation.

The translated genomes of Streptococcus agalactiae strain CJB111 (RefSeq assembly accession no. GCF_015221735.2) and Akkermansia muciniphila *muc* strain (RefSeq assembly accession no. GCF_000020225.1) were downloaded from NCBI. CarveMe was used for the fast, automated reconstruction of genome-scale metabolic models of each genome under the appropriate Gram-positive or Gram-negative template (21). The resulting metabolic models were treated as an input microbial community to SMETANA, which computes metrics that describe potential cross-feeding interactions (20, 22). Metrics include metabolic resource overlap (MRO), species coupling score (SCS), metabolite uptake score (MUS), metabolite production score (MPS), and SMETANA score. MRO is a method of assessing metabolic competition by measuring the overlap between the minimal nutritional requirements of all member species based on their genomes (22). SCS is a community size-dependent measurement of the dependency of one species in the presence of the others to survive. MUS measures how frequently a species needs to uptake a metabolite to survive. MPS is a binary measurement of the ability of a species to produce a metabolite. The individual SMETANA score is a combination of the SCS, MUS, and MPS scores and gives a measure of certainty on a cross-feeding interaction (e.g., species A receives metabolite X from species B). Simulations were created based on a minimal media calculated using molecular weights that supports the growth of both organisms with the inorganic compounds of hydrogen, water, and phosphate excluded from analysis.

### GBS and *A. muciniphila* detection within a human cohort.

Human vaginal microbiome raw data was obtained from Callahan et al. (19) under SRA accession no. SRP115697. This data set was processed in a similar manner as murine microbiome samples described above. Briefly, paired-end reads were imported into QIIME2, demultiplexed, and merged with VSEARCH (33). Joined reads were quality filtered and denoised with Deblur to generate suboperational taxonomic units (i.e., microbial features) and trimmed to 250 bp (34). The Greengenes 13.8 99% OTU database was utilized for phylogenetic tree creation with SEPP fragment insertion and for taxonomic assignment with a naïve Bayes classifier trained on the 16S rRNA V4 region (35, 36). Microbial features were filtered out if they were assigned to mitochondria, chloroplast, or not of bacterial origin. Further, features were removed to reduce noise if present less than 10 times in the data set and/or not found in at least 2 samples. Samples with less than 40,000 features were removed to ensure we included high-depth samples. After filtering, the data set includes 2,110 samples and 3,470 unique features. Detection of GBS and *A. muciniphila* in the term and preterm birth groups was determined by the presence/absence of a species at any time point during pregnancy.

10.1128/msphere.00264-22.2 FIG S2 Linear mixed-effects models for measuring the impacts of time and treatment on the CLR-transformed abundances of Staphylococcus (A), *Enterobacteriaceae* (B), *Enterococcus* (C), Proteus *sp*. (D), Bifidobacterium pseudolongum (E), *Lactobacillus sp*. (F), Lactobacillus salivarius (G), *Bacillus sp*. (H), *Ruminococcaceae sp*. (I), *Corynebacterium sp*. (J), Streptococcus (K), Morganella morganii (L), and *Enterococcus sp.* (M). Download FIG S2, TIF file, 1.4 MB. Copyright © 2022 Burcham et al. 2022 Burcham et al. https://creativecommons.org/licenses/by/4.0/ This content is distributed under the terms of the Creative Commons Attribution 4.0 International license.
