# Supplementary figures and images for: Interrelated Effects of Zinc Deficiency and the Microbiome on Group B Streptococcal Vaginal Colonization
Source: mSphere. 2022 Aug 9;7(4):e00264-22. doi: 10.1128/msphere.00264-22 (PMC9429885; doi:10.1128/msphere.00264-22)

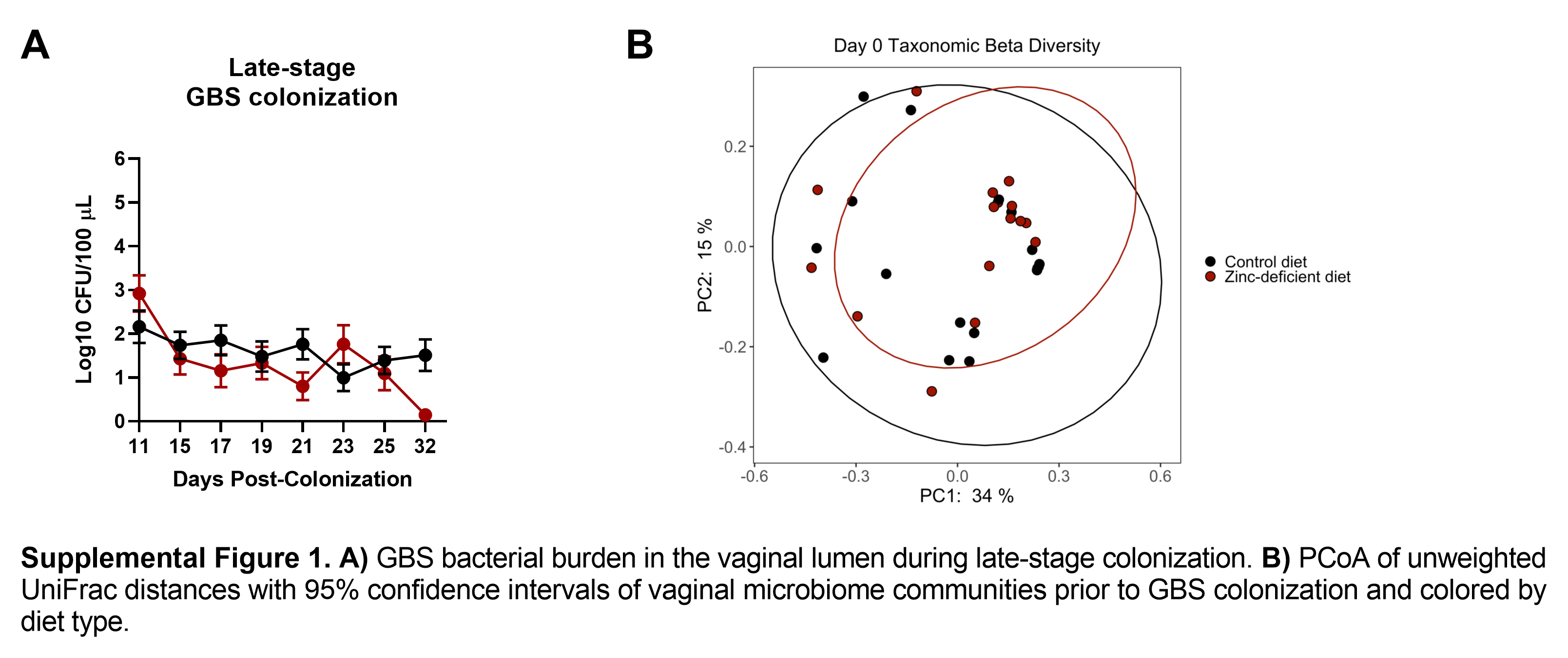

Supplement: FIG S1 [file msphere.00264-22-s0001.tif]

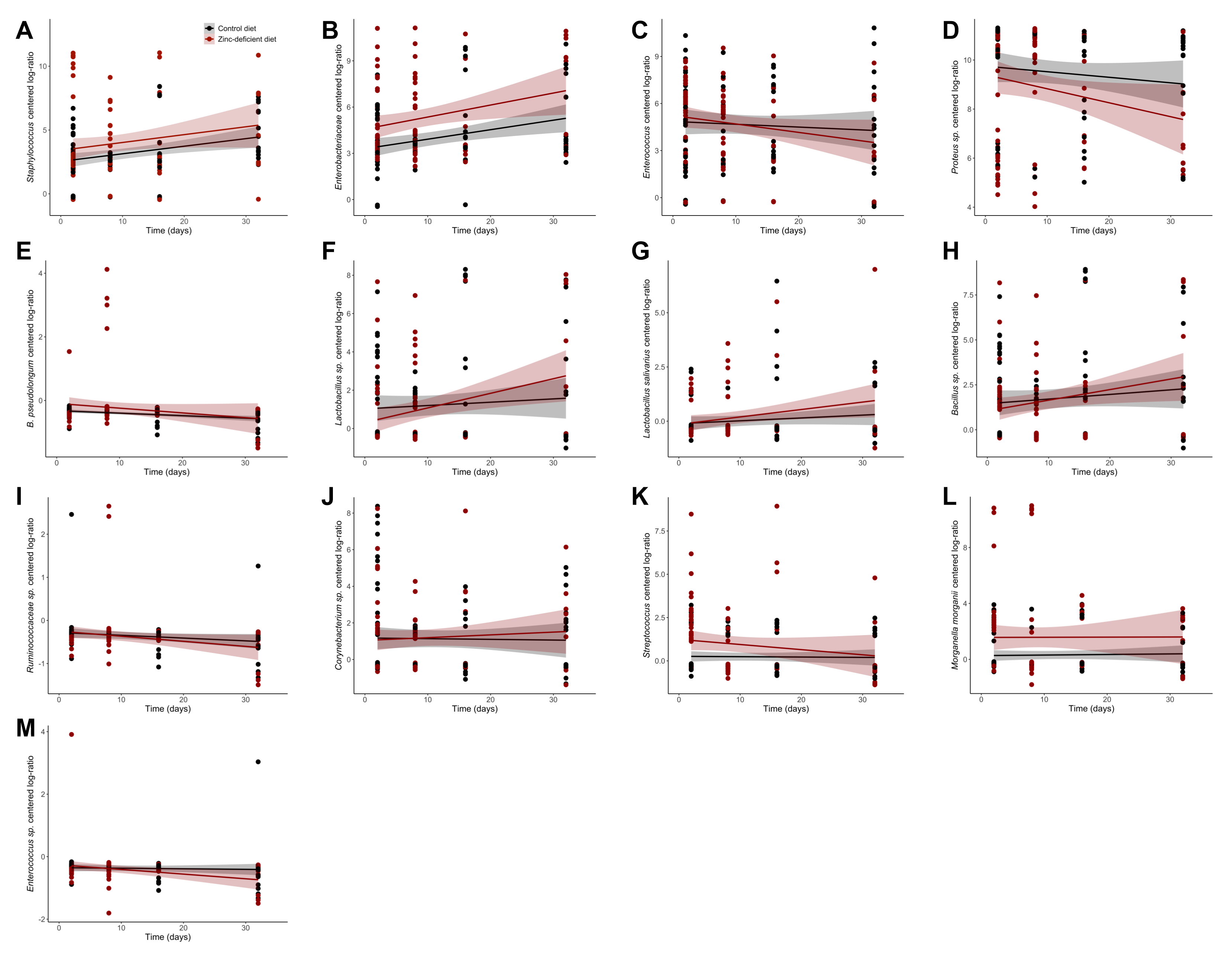

Supplement: FIG S2 [file msphere.00264-22-s0002.tif]
